# Cribado de tuberculosis en jóvenes migrantes procedentes de Mali: concordancia entre Mantoux y QuantiFERON® en Atención Primaria

**DOI:** 10.1016/j.aprim.2026.103489

**Published:** 2026-05-13

**Authors:** Íñigo Hidalgo Rípodas, Arantxa Elizalde Sanabria

**Affiliations:** C.S. Buztintxuri, Servicio Navarro de Salud, Pamplona, Navarra, España

La migración irregular desde Mali hacia España ha experimentado un aumento notable en los últimos años, pasando de cifras cercanas a las 2.000 llegadas en 2023 a superar las 10.000 entre 2024 y 2025. Esta tendencia ascendente, impulsada en gran medida por la inestabilidad en la región del Sahel, hace indispensable conocer con profundidad la situación epidemiológica de este colectivo para implementar un cribado sanitario eficaz. Mali presenta una alta incidencia de tuberculosis (TB) (48 casos por 100.000 habitantes)^1^ y mantiene una política de vacunación sistemática con bacilo de Calmette-Guérin (BCG). La realidad de la comunidad autónoma del estudio refleja tasas de TB significativamente superiores en población migrante respecto a la autóctona, con 35 frente a 4 casos por cada 100.000 habitantes.

En el ámbito cotidiano de la Atención Primaria, el test de Mantoux (TST) suele ser la primera prueba de cribado para la infección tuberculosa latente (ITL). Sin embargo, su especificidad se reduce considerablemente en población vacunada con BCG^2^, generando reactividad inespecífica y falsos positivos que derivan en pruebas complementarias innecesarias. El objetivo de este trabajo fue evaluar el rendimiento diagnóstico del TST frente al ensayo QuantiFERON (QFT) en varones jóvenes malienses y analizar las limitaciones operativas del protocolo vigente.

Realizamos un estudio observacional, transversal y descriptivo retrospectivo de los registros del cribado efectuado durante 2025 en un centro de salud del norte de España. Incluimos 55 varones refugiados con una edad media de 25,5 años, obteniendo un análisis válido de ITL en 44 participantes tras excluir casos por rechazo a la técnica o a la lectura. Para la elaboración de este artículo, declaramos que se han seguido los protocolos del centro de trabajo sobre la publicación de datos de pacientes y se ha respetado la privacidad de los sujetos. El protocolo actual indica TST inicial y confirmación con QFT en induraciones de 5 a 14 mm; los resultados ≥ 15 mm se consideran directamente ITL y se derivan a radiografía de tórax (RTx).

La prevalencia de ITL fue del 61,4%. Debido a una desviación incidental del protocolo (error administrativo), se realizó QFT a 12 participantes con TST ≥ 15 mm. De ellos, 7 resultaron negativos (58,3%), lo que indica una elevada proporción de falsos positivos en este rango de induración (fig. 1, panel A). En este subgrupo, el TST ≥ 15 mm mostró un valor predictivo positivo del 41,7% y una concordancia pobre con el QFT, con un índice Kappa de 0,10. No hallamos asociación estadísticamente significativa entre el tamaño de la induración y el resultado del QFT mediante la prueba U de Mann-Whitney (p = 0,245).

**Figura 1:**
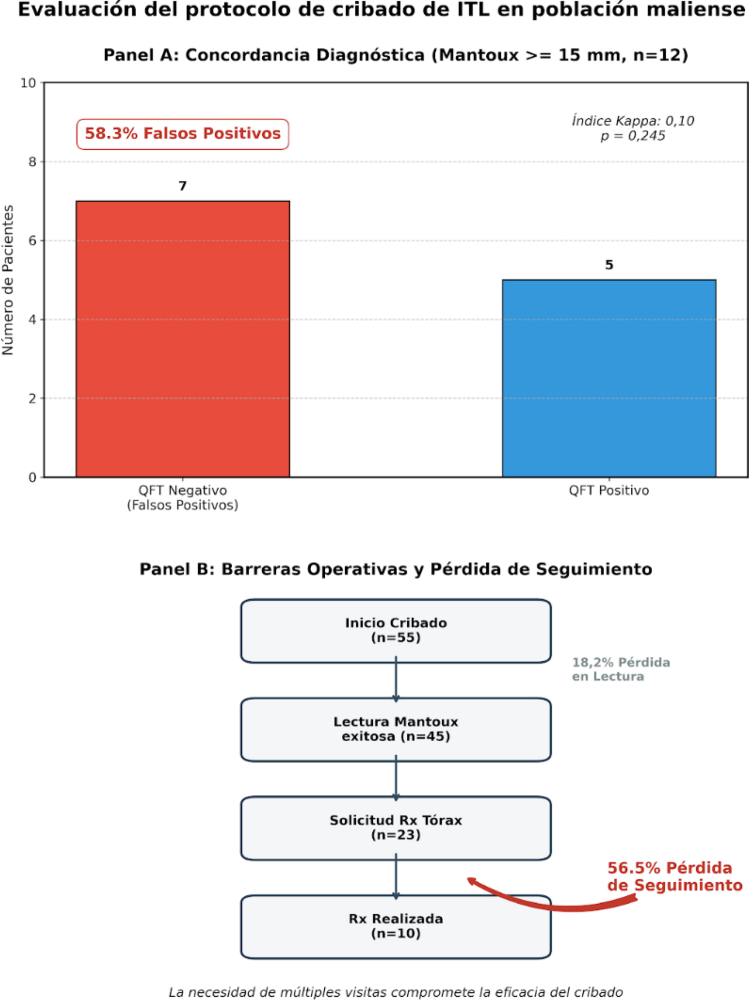
Análisis del rendimiento diagnóstico y efectividad operativa del protocolo de cribado.Panel A: Distribución de resultados de QuantiFERON (QFT) en sujetos con test de Mantoux (TST) ≥ 15 mm (n = 12). Se observa un 58,3% de falsos positivos y una baja concordancia entre ambas pruebas (índice Kappa: 0,10; p = 0,245).Panel B: Cascada de seguimiento clínico desde el inicio del cribado (n = 55) hasta la realización de la radiografía de tórax (n = 10). Se destaca una pérdida de seguimiento del 18,2% en la fase de lectura y una pérdida crítica del 56,5% en la fase radiológica. La necesidad de múltiples visitas físicas actúa como el principal obstáculo operativo para completar el proceso diagnóstico.

Adicionalmente, se solicitaron 23 radiografías para descartar TB activa. Observamos una tasa de pérdida de seguimiento del 56,5% por varios participantes que no completaron la prueba radiológica (fig. 1, panel B). Ninguna de las placas realizadas mostró signos de enfermedad activa.

Nuestros resultados muestran que el TST tiene una capacidad predictiva limitada en jóvenes vacunados con BCG. Nuestros hallazgos de baja concordancia (Kappa 0,10) refuerzan la evidencia sobre la interferencia de la vacuna BCG en la interpretación del TST. Asimismo, la necesidad de múltiples visitas supone un obstáculo operativo crítico en una población con alta movilidad, contribuyendo al abandono del proceso diagnóstico (fig. 1, panel B)^3^.

Estos datos sugieren que el protocolo de cribado en Atención Primaria debería optimizarse. Derivar sistemáticamente a QFT a los pacientes con TST ≥ 5 mm, tal como recomiendan organismos internacionales y la normativa de la SEPAR^4^, ^5^ para migrantes de áreas de alta incidencia, permitiría reducir pruebas innecesarias, mejorar la fiabilidad diagnóstica y disminuir la pérdida de seguimiento.

## Consideraciones éticas

Se contó con aprobación del CEIm-NA y se respetó la Declaración de Helsinki y la privacidad de los pacientes. Se obtuvo consentimiento informado según protocolos institucionales.

## Declaración sobre el uso de la IA generativa y de las tecnologías asistidas por la IA en el proceso de redacción

Se utilizó inteligencia artificial generativa únicamente para mejorar la redacción del texto. El análisis de datos y las conclusiones son responsabilidad exclusiva de los autores.

## Financiación

Los autores declaran que no se recibió financiación externa para la realización del estudio. La publicación de este artículo en acceso abierto ha sido financiada íntegramente por la Fundación Miguel Servet-Navarrabiomed (CIF ES G31187420) a través de su convocatoria de ayudas para la difusión de resultados de investigación.

## Conflicto de intereses

Los autores declaran no tener conflictos de intereses.
